# Characterizing chronological accumulation of comorbidities in healthy veterans: a computational approach

**DOI:** 10.1038/s41598-021-85546-2

**Published:** 2021-04-14

**Authors:** Julian C. Hong, Elizabeth R. Hauser, Thomas S. Redding, Kellie J. Sims, Ziad F. Gellad, Meghan C. O’Leary, Terry Hyslop, Ashton N. Madison, Xuejun Qin, David Weiss, A. Jasmine Bullard, Christina D. Williams, Brian A. Sullivan, David Lieberman, Dawn Provenzale

**Affiliations:** 1 Cooperative Studies Program Epidemiology Center-Durham, Durham VA Health Care System, Durham, NC USA; 2 Department of Radiation Oncology, University of California, San Francisco, San Francisco, CA USA; 3 Bakar Computational Health Sciences Institute, University of California, San Francisco, San Francisco, CA USA; 4 Department of Biostatistics and Bioinformatics, Duke University, Durham, NC USA; 5 Department of Medicine, Duke University, Durham, NC USA; 6 Cooperative Studies Program Coordinating Center, Perry Point VA Medical Center, Perry Point, MD USA; 7 VA Portland Health Care System, Portland, OR USA; 8 Oregon Health and Science University, Portland, OR USA

**Keywords:** Data mining, Comorbidities

## Abstract

Understanding patient accumulation of comorbidities can facilitate healthcare strategy and personalized preventative care. We applied a directed network graph to electronic health record (EHR) data and characterized comorbidities in a cohort of healthy veterans undergoing screening colonoscopy. The Veterans Affairs Cooperative Studies Program #380 was a prospective longitudinal study of screening and surveillance colonoscopy. We identified initial instances of three-digit ICD-9 diagnoses for participants with at least 5 years of linked EHR history (October 1999 to December 2015). For diagnoses affecting at least 10% of patients, we calculated pairwise chronological relative risk (RR). iGraph was used to produce directed graphs of comorbidities with RR > 1, as well as summary statistics, key diseases, and communities. A directed graph based on 2210 patients visualized longitudinal development of comorbidities. Top hub (preceding) diseases included ischemic heart disease, inflammatory and toxic neuropathy, and diabetes. Top authority (subsequent) diagnoses were acute kidney failure and hypertensive chronic kidney failure. Four communities of correlated comorbidities were identified. Close analysis of top hub and authority diagnoses demonstrated known relationships, correlated sequelae, and novel hypotheses. Directed network graphs portray chronologic comorbidity relationships. We identified relationships between comorbid diagnoses in this aging veteran cohort. This may direct healthcare prioritization and personalized care.

## Introduction

Multimorbidity and chronic comorbidity have negative consequences on health outcomes, quality of life, and costs^1,2^. Therefore, understanding health trajectory and the accumulation of comorbidities is critical to better characterizing and potentially mitigating subsequent disease processes. Anticipating comorbidities at the individual-level may direct clinicians towards appropriate preventative strategies^3^. On a population basis, understanding the trajectory of diseases can direct utilization of resources to prevent downstream comorbidities.

Many advances have been made in characterizing environmental, genomic, and proteomic etiologies of disease. Diseases represent a complex network of conditions, with a variety of causal and correlated temporal relationships. Computational methods have also begun to take advantage of the vast clinical histories available in claims data to characterize longitudinal associations between comorbidities and/or patient phenotypes both in the form of undirected social networks^4–12^ and clusters^13–15^. Widespread use of electronic health records (EHR) can further facilitate this detailed characterization of disease states and trajectories.

Prior studies have previously demonstrated the feasibility of representing the progression of disease through directed networks^16–19^. These networks provide the advantage of incorporating the chronicity of comorbidities, which is fundamental to understanding potential progression of disease and patient health trajectories. Findings from temporal co-occurrence using EHR data have previously reported on complications from specific diagnoses^19–22^, racial variation^23^, and inpatient trajectories^24^. In the veteran population, prior studies have focused network analyses on mental health or described limited clusters in veterans with specific service histories^10,14^. Expanding these approaches to include a larger array of comorbidities and trajectories in an initially healthy population may facilitate screening, preventative care, or early intervention in the longitudinal clinical setting, and also generate new hypotheses for exploration.

It is particularly salient from a healthcare delivery standpoint to optimize care within the Veterans Health Administration (VHA), which is the largest integrated healthcare system in the United States. Understanding comorbidities is especially important among the veteran population, as comorbidities may be more prevalent and severe than in the general population and vary based on military service history. The Veterans Affairs (VA) Cooperative Studies Program (CSP) #380 study is a prospective longitudinal study of screening and surveillance colonoscopy in a healthy cohort of asymptomatic veterans enrolled from 1994–1997^25,26^. Multiple data sources have been linked in this cohort, enriching the prospectively collected study data with additional resources such as the EHR. These robust longitudinal data over long-term follow-up offer a unique opportunity to generate a better understanding of patterns of disease in a well-defined and initially healthy cohort.

The objective of this study is to apply network analysis to the well-selected and prospectively identified CSP #380 screening colonoscopy cohort to characterize the longitudinal sequence of diagnoses using a directed graph. This approach can be used to summarize the development of diseases in this cohort and developed as a framework to provide visual tools to guide clinicians in understanding potential downstream diseases for patients.

## Results

Of the 3121 CSP #380 participants, 2787 patients had linked data in the CDW, with 2210 having at least 5 years between their earliest and latest encounters from October 1999 to December 2015 (Table 1 and Supplementary Table S1).

**Table 1:** Patient characteristics (n = 2210).

Variable	Number (%)/Median (IQR)
Male	2132 (96.5%)
**Age**	
At first diagnosis (years)	67.2 (60.4–72.2)
At last diagnosis (years)	79.4 (73.6–84.5)
Years of follow-up	14.4 (9.6–15.8)
EHR ICD diagnoses per patient	392.0 (214.0–696.5)
Number of distinct ICD diagnoses	95 (60–138)
Number of distinct ICD three-digit diagnoses	64 (43–86)

*EHR* Electronic health record, *ICD* International Classification of Diseases (ninth edition).

*Among 3,121 patients included in CSP #380, 2,787 had available data with 2,210 having 5-year follow-up.

### Patient and diagnosis characteristics

Patients had a median first diagnosis at age 67.2 and last diagnosis at 79.4 (Table 1). The median diagnosis history was 14.4 years (range 5.0–16.2 years). Patients had a median of 95 distinct ICD-9 diagnoses, representing 64 distinct three-digit diagnoses.

Among three-digit ICD-9 codes, the most common codes included a variety of non-diagnostic V codes (Supplementary Classification of Factors Influencing Health Status and Contact with Health Services) (Table 2). The most common diagnostic codes included essential hypertension (401.*), disorders of lipid metabolism (272.*), general symptoms (780.*), symptoms involving the respiratory system (786.*), and cataracts (366.*). The most common non-diagnostic codes were other persons seeking consultation (V65.*), need for prophylactic vaccination and inoculation (V04.*), encounters for administrative purposes (V68.*), special investigations and examinations (72.*), and encounter for other and unspecified procedures and aftercare (V58.*).

**Table 2:** Most common three-digit ICD diagnoses by EHR coding among CSP #380 participants (n = 857).

ICD-9	Diagnosis	# encounters	# patients	% patients
V65.*	Other persons seeking consultation	51,916	2122	96.0
401.*	Essential hypertension	64,917	1999	90.5
V04.*	Need for prophylactic vaccination and inoculation against certain diseases	14,014	1954	88.4
272.*	Disorders of lipoid metabolism	33,663	1803	81.6
V68.*	Encounters for administrative purposes	16,087	1737	78.6
780.*	General symptoms	17,167	1678	75.9
V72.*	Special investigations and examinations	10,712	1624	73.5
786.*	Symptoms involving respiratory system and other chest symptoms	15,210	1615	73.1
366.*	Cataract	14,733	1580	71.5
367.*	Disorders of refraction and accommodation	13,642	1563	70.7
719.*	Other and unspecified disorders of joint	12,373	1531	69.3
V58.*	Encounter for other and unspecified procedures and aftercare	37,390	1520	68.8
V70.*	General medical examination	5408	1392	63.0
715.*	Osteoarthrosis and allied disorders	17,679	1376	62.3
V57.*	Care involving use of rehabilitation procedures	25,701	1353	61.2
724.*	Other and unspecified disorders of back	16,596	1292	58.4
782.*	Symptoms involving skin and other integumentary tissue	5697	1275	57.7
V81.*	Special screening for cardiovascular, respiratory, and genitourinary diseases	8361	1249	56.5
600.*	Hyperplasia of prostate	10,435	1204	54.5
389.*	Hearing loss	11,075	1191	53.9
530.*	Diseases of esophagus	11,930	1175	53.2
427.*	Cardiac dysrhythmias	31,419	1136	51.4
702.*	Other dermatoses	9129	1126	51.0
211.*	Benign neoplasm of other parts of digestive system	4203	1124	50.9
414.*	Other forms of chronic ischemic heart disease	21,975	1105	50.0

Includes all diagnoses among patients in the VA Corporate Data Warehouse.

### Network characterization

**Figure 1:**
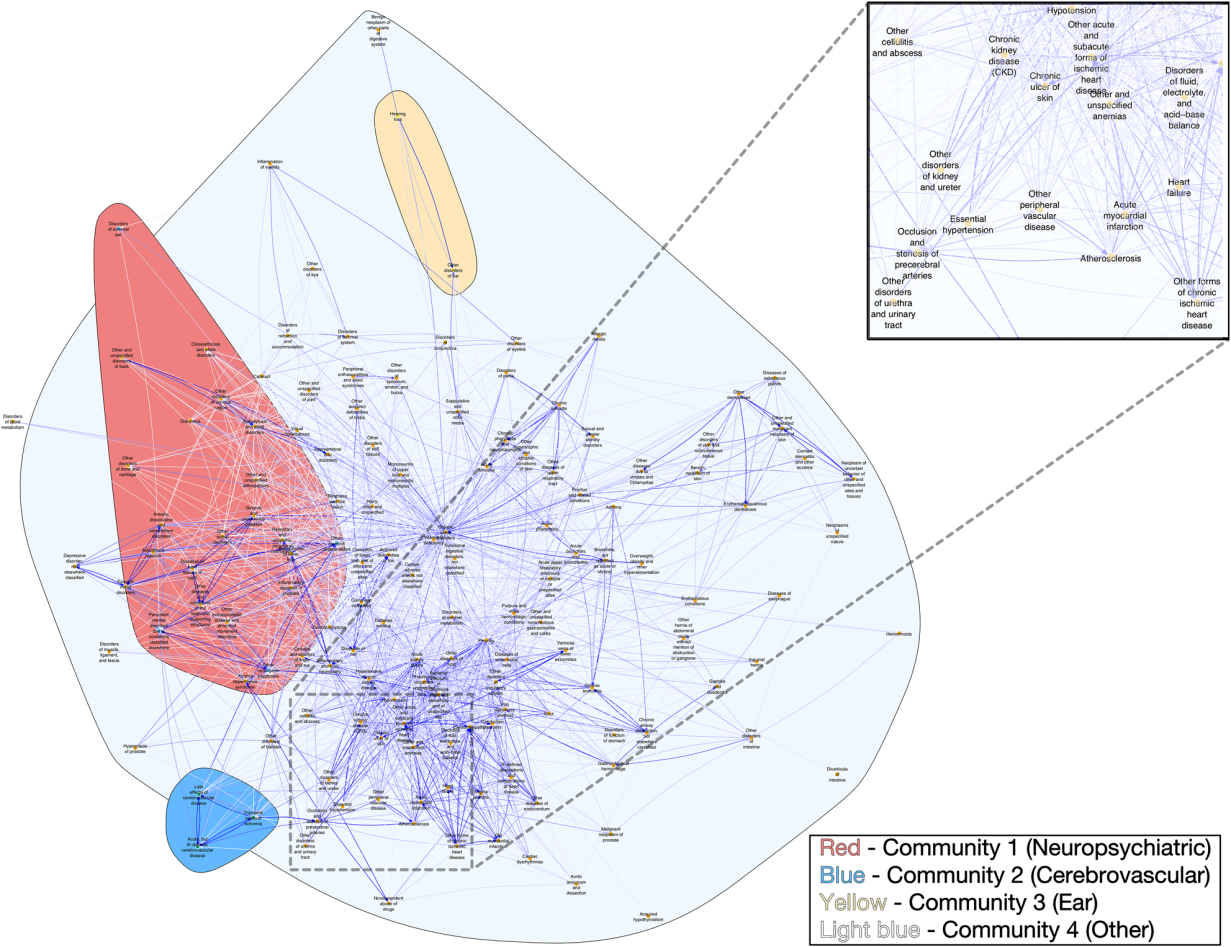
Diagnosis directed network. Darker connections indicate greater relative risk relationships. Diagnoses clustered into four communities: neuropsychiatric disorders, cerebrovascular disorders, ear disorders, and all others. Sample diagnoses visible in inset. Zoomable image is available online.

To describe the progression of comorbidities in this cohort of veterans, we generated a disease network of diagnoses affecting at least 10% of patients, excluding non-diagnostic V codes and symptoms, signs, and ill-defined conditions (total of 145 distinct ICD three-digit codes; Fig. 1). Important characteristics of the network included edge density with 11% of the possible chronological relationships represented in the network and reciprocity with 23% of the pairwise relationships bidirectional. These measures indicate a limited number of potential pairwise chronological relationships and bidirectional relationships. Key diseases in the graph were also identified, including hubs and authorities. Top hub (preceding) diseases included ischemic heart disease (411.*), inflammatory and toxic neuropathy (357.*), and diabetes mellitus (250.*) (Table 3). Top authority (subsequent) diagnoses were acute kidney failure (584.*), hypertensive chronic kidney disease (403.*), and pleurisy (511.*) (Table 4). The top 25 PageRank diagnoses largely mirrored authority diagnoses in aggregate though the three highest scoring conditions were acute kidney failure (584.*), vitamin D deficiency (268*), and bacterial infection in conditions classified elsewhere and of unspecified site (041.*) (Table 5). Several diagnoses had high hub, authority, and PageRank scores, including acute kidney failure (584.*), iron deficiency anemia (280.*), fluid, electrolyte, and acid–base balance disorders (276.*), and chronic kidney disease (585.*).

**Table 3:** Highest hub score diagnosis codes (n = 142).

ICD-9	Diagnosis	Hub score
411.*	Other acute and subacute forms of ischemic heart disease	1.00
**357**.*	**Inflammatory and toxic neuropathy**	**0.88**
250.*	Diabetes mellitus	0.86
703.*	Diseases of nail	0.82
**585**.*	**Chronic kidney disease (CKD)**	**0.78**
440.*	Atherosclerosis	0.75
**425**.*	**Cardiomyopathy**	**0.75**
429.*	Ill-defined descriptions and complications of heart disease	0.74
428.*	Heart failure	0.71
110.*	Dermatophytosis	0.69
401.*	Essential hypertension	0.68
700.*	Corns and callosities	0.68
413.*	Angina pectoris	0.65
**410**.*	**Acute myocardial infarction**	**0.65**
593.*	Other disorders of kidney and ureter	0.64
**280**.*	**Iron deficiency anemias**	**0.63**
274.*	Gout	0.63
**276**.*	**Disorders of fluid, electrolyte, and acid–base balance**	**0.63**
414.*	Other forms of chronic ischemic heart disease	0.63
443.*	Other peripheral vascular disease	0.59
**584**.*	**Acute kidney failure**	**0.55**
	Hypotension	0.54
424.*	Other diseases of endocardium	0.53
	Chronic ulcer of skin	0.53

Bold denotes diagnoses that are among both top hubs and authorities.

**Table 4:** Highest authority score diagnosis codes (n = 142).

ICD-9	Diagnosis	Authority score
**584**.*	**Acute kidney failure**	**1.00**
403.*	Hypertensive chronic kidney disease	0.90
511.*	Pleurisy	0.84
327.*	Organic sleep disorders	0.82
275.*	Disorders of mineral metabolism	0.64
041.*	Bacterial infection in conditions classified elsewhere and of unspecified site	0.64
458.*	Hypotension	0.64
**280**.*	**Iron deficiency anemias**	**0.59**
518.*	Other diseases of lung	0.55
268.*	Vitamin D deficiency	0.53
**276**.*	**Disorders of fluid, electrolyte, and acid–base balance**	**0.52**
**585**.*	**Chronic kidney disease (CKD)**	**0.51**
**425**.*	**Cardiomyopathy**	**0.48**
285.*	Other and unspecified anemias	0.47
486.*	Pneumonia, organism unspecified	0.46
288.*	Diseases of white blood cells	0.42
707.*	Chronic ulcer of skin	0.41
331.*	Other cerebral degenerations	0.40
287.*	Purpura and other hemorrhagic conditions	0.40
426.*	Conduction disorders	0.38
298.*	Other nonorganic psychoses	0.37
**357**.*	**Inflammatory and toxic neuropathy**	**0.36**
	Contusion of lower limb and of other and unspecified sites	0.34
**410**.*	**Acute myocardial infarction**	**0.35**

Bold denotes diagnoses that are among both top hubs and authority.

**Table 5:** Highest PageRank diagnosis codes (n = 142).

ICD-9	Diagnosis	PageRank
**584**.*	**Acute kidney failure**	**0.062**
**268**.*	**Vitamin D deficiency**	**0.048**
**041**.*	**Bacterial infection in conditions classified elsewhere and of unspecified site**	**0.047**
**511**.*	**Pleurisy**	**0.047**
**275**.*	**Disorders of mineral metabolism**	**0.045**
294.*	Persistent mental disorders due to conditions classified elsewhere	0.039
**331**.*	**Other cerebral degenerations**	**0.038**
**403**.*	**Hypertensive chronic kidney disease**	**0.036**
**285**.*	**Other and unspecified anemias**	**0.031**
**276**.*	**Disorders of fluid, electrolyte, and acid–base balance**	**0.031**
**458**.*	**Hypotension**	**0.030**
**280**.*	**Iron deficiency anemias**	**0.029**
**486**.*	**Pneumonia, organism unspecified**	**0.028**
**327**.*	**Organic sleep disorders**	**0.027**
**518**.*	**Other diseases of lung**	**0.027**
**288**.*	**Diseases of white blood cells**	**0.024**
**287**.*	**Purpura and other hemorrhagic conditions**	**0.022**
995.*	Certain adverse effects not elsewhere classified	0.022
**707**.*	**Chronic ulcer of skin**	**0.017**
**585**.*	**Chronic kidney disease (CKD)**	**0.016**
**298**.*	**Other nonorganic psychoses**	**0.016**
**425**.*	**Cardiomyopathy**	**0.012**
924.*	Contusion of lower limb and of other and unspecified sites	0.010
410.*	Acute myocardial infarction	0.009
491.*	Chronic bronchitis	0.007

Bold denotes diagnoses that are among both top authorities and PageRank.

**Figure 2:**
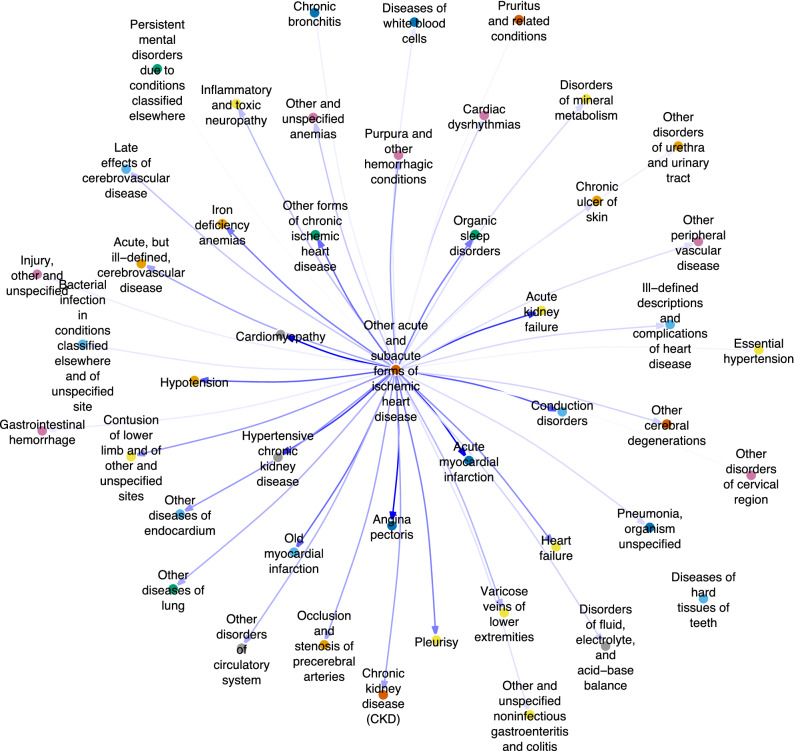
Directed network graph ICD 411.* (Other acute and subacute forms of ischemic heart disease) and highest relative risk subsequent diagnoses. ICD 411.* was identified as a major hub diagnosis, with important subsequent diagnoses shown below. A broad range of diagnoses form the network, including clinically anticipated diagnoses (cardiac and renal diseases) and those that share etiologies (vascular). Others may share less apparent common etiologies (smoking and lung disease) or may be the result of intermediary comorbidities (anemia due to renal disorders).

We more closely investigated the diagnoses with the top hub and authority scores to better contextualize their comorbiditiy. ICD 411.* (other acute and subacute forms of ischemic heart disease) had the highest hub score, and commonly preceded a number of expected diagnoses, including cardiac (acute myocardial infarction, cardiomyopathy) and renal (acute kidney failure, hypertensive chronic kidney disease) diagnoses (Supplementary Table S2; Fig. 2). Other subsequent diagnoses to ischemic heart disease included those with similar vascular etiologies (e.g., occlusion and stenosis of precerebral arteries and acute cerebrovascular disease). Other high relative risk diagnoses included organic sleep disorders, iron deficiency anemia, other diseases of the lung and pleurisy. These may share more distant causal etiologies such as smoking (lung disease) or be indirectly related due to intermediary comorbidities (e.g., renal disorders resulting in anemia).

**Figure 3:**
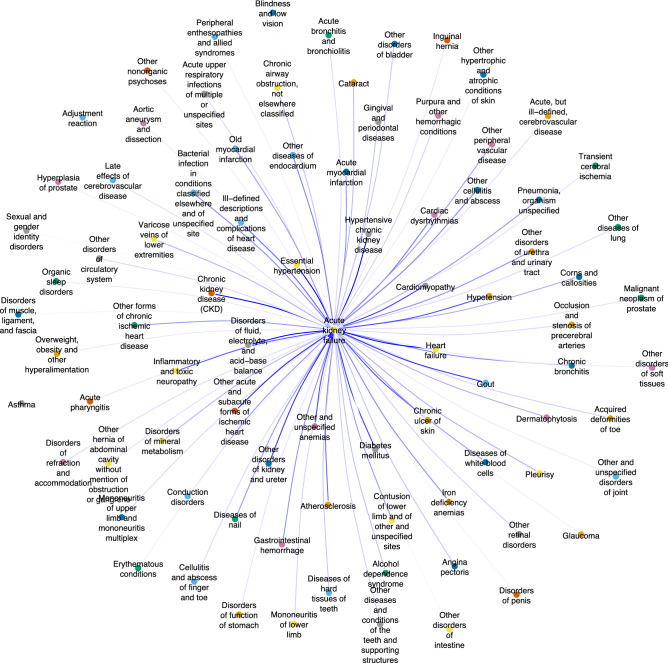
Directed network graph of diagnoses with greatest relative risk of subsequent acute kidney failure (ICD 584.*). Acute kidney failure was identified as a major authority diagnosis, with important preceding diagnoses shown below. A broad range of diagnoses form the network, including clinically anticipated precursor diagnoses (hypertension, cardiac disease, renal disease, and diabetes), correlated renal sequelae (anemia, electrolyte disorders), and less clinically anticipated preceding diagnoses (gout, skin ulcers).

Acute kidney failure (ICD 584.*) had the highest authority and PageRank scores, representing one of the most common subsequent diseases. Diagnoses which carried a high relative risk for subsequent diagnosis of acute included expected diagnoses such as essential hypertension, chronic renal dysfunction (chronic kidney disease, hypertensive chronic kidney disease, other disorders of kidney and ureter), cardiac disease (heart failure, ischemic heart disease), and diabetes (Supplementary Table S3; Fig. 3). Correlated sequelae of chronic renal dysfunction such as electrolyte disorders and anemia were also observed. Finally, a number of less clinically anticipated diagnoses carried a high relative risk of subsequent acute kidney failure, including gout, chronic ulcer of the skin, and inflammatory and toxic neuropathy.

The greatest RR relationships were used to generate sample diagnosis paths from the highest hub diagnosis, other acute and subacute forms of ischemic heart disease (ICD 411.*), demonstrating a rational pathway to chronic pulmonary heart disease (ICD 416.*) (Supplementary Figure S1). Similarly, the greatest RR relationships were investigated to create a path to acute kidney failure (ICD 584.*), which had the highest authority score. This demonstrated a progression from diabetes mellitus (ICD 250.*) with aggregation of other diabetic complications (neuropathy, hypertension) before reaching acute kidney failure.

Infomap identified four communities of diagnoses (Fig. 1; Supplementary Table S4). One was primarily neuropsychiatric, including disorders of the inner ear (380.*), persistent mental disorders due to conditions classified elsewhere (294.*), other nonorganic psychoses (298.*), and other cerebral degenerations (331.*). The second was cerebrovascular in nature, with acute, but ill-defined cerebrovascular disease (436.*), late effects of cerebrovascular disease (438.*), and transient cerebral edema (435.*). A third community included ear disorders, with hearing loss (389.*) and other disorders of the ear (388.*), and the fourth one included all other diagnoses.

## Discussion

This study demonstrates the usability of directed graphs built on longitudinal EHR-based data to characterize disease trajectories and demonstrate expected and unexpected relationships between comorbidities in a prospective veteran cohort. To date, there are limited reports on the application of directed graphs using routinely-collected EHR data^16,19^. We applied this methodology specifically to EHR data for a well-defined, prospectively followed homogeneous, initially healthy, aging veteran population, and identified key diagnoses in comorbidity trajectories and clusters of interrelated diagnoses. These can have applications in the individual and broader healthcare delivery levels.

On the individual level, characterizing health and disease trajectories is crucial to understand the aging process and the aggregation of comorbidities over time. Studies of resilience and frailty in the field of aging rely on the ability to develop individual-level multi-component trajectories^27,28^. We were able to leverage network analysis to understand patterns of diagnoses, which may allow the anticipation of future multi-morbidity. In practice, a patient’s constellation of ICD diagnoses could be populated in an automated fashion from the EHR to achieve two potentially clinically relevant objectives. First, hub diagnoses (those which carry high subsequent multi-morbidity) may be systematically flagged for aggressive management. Many of these are already important to clinicians: heart disease, diabetes, renal disorders, but quantification of their importance in accumulating comorbidities can both more objectively guide clinicians and enhance counseling for patients. Second, this approach can use existing conditions to identify potential subsequent diseases to guide screening, prevention, and management strategies. For instance, a patient with diagnoses of hypertension and heart failure would be anticipated to have high risk for chronic kidney disease. Network analyses may automatically recognize these preceding EHR diagnoses to provide reminders for preventative strategies such as use of ACE inhibitors when appropriate. Additionally, our network approach offers the benefit of both synthesizing multiple potential precursors to potential diagnoses, as well as identifying less clinically obvious diagnoses which may signal future risk, such as gout or skin ulcers. Our network enables identification of diagnoses multiple steps downstream, such as ischemic heart disease leading to renal failure leading to anemia. Preventative strategies, at earlier points in these disease paths should be considered to prevent future comorbidity.

These data can also be generalized to a cohort or practice setting and inform healthcare priorities. The CSP #380 veteran participants evaluated in this study are representative of aging patients who were generally healthy at the start of their enrollment in the study, allowing characterization of this VHA population over time. The status of their health at the initiation of the study was prospectively verified, and due to their enrollment on study, have a higher rate of follow-up than would be anticipated from the general population, making this cohort well-suited for this analysis. Our network analysis in the VHA is unique, in that it followed patient trajectories in the largest integrated healthcare system in the United States and could provide insight to develop streamlined care pathways or prioritize high impact diagnoses. In our cohort, both acute and chronic renal diagnoses were among those with highest authority and PageRank scores, two confirmatory approaches to identify common subsequent diagnoses. Their identification by these metrics suggest that their downstream complications or subsequent associated comorbidities may have wide-reaching impacts in this specific cohort. Aggressive screening^29^, prediction^30^, and appropriate clinical management of renal disorders^31,32^ have been areas of active investigation in the VHA and represent areas for prioritization. Future work could leverage network analyses to improve efficacy and efficiency in a health care setting of potentially constrained resources.

Community detection enables the partitioning of diagnoses that are more densely connected to each other than with the rest of the network. This can enable an understanding of potential disease pathways of interrelated diagnoses, much like protein interaction networks can reveal shared functionalities^27^. In our initially healthy VHA cohort, we identified communities that appeared to be clinically rational, representing neuropsychiatric, cerebrovascular, and hearing disorders. Each community represents diseases that tend to coexist in patients in greater isolation from other diagnoses. It is possible that for certain cohorts, these might direct the design of teams to comanage a patient who has comorbidities within a single network community.

Our study is unique among prior studies investigating applications of undirected networks to temporal diagnoses^16–19^ due to its comprehensive nature as a primary EHR analysis in initially healthy patients who were seen longitudinally in a single health system (median of 14.4 years). While prior studies of this type have not reported the duration of follow-up, an extensive study of the Danish National Patient Registry (NPR) analyzed patients over a 14.9-year period with likely complete data given mandatory reporting^18^. Diverse approaches have been used across the prior studies, particularly around the methodology for assessing relationships between diagnosis pairs. These have included the use of relative risk^17,18^ and binomial test p-values^16,19^. We opted for the use of relative risk in this study given its intuitive nature for clinicians. Other nuances to minimize false positives have been discordant across studies, including the use of prevalence^16^ and relative risk^17^ criteria. As both attempt to limit relationships that may be included due to rare diagnoses, we chose to limit our network to diagnoses that were present in at least 10% of the population to not exclude truly high RR relationships. Finally, given its strength in community detection across different network sizes^33^ and its prior use in a study utilizing claims data^17^, we opted to use the Infomap algorithm for community detection.

Using the top hub and authority diagnoses as case studies, we were able to confirm well-known relationships (like cardiac and renal comorbidity), but also characterize correlated diagnoses of clearly shared etiology (acute kidney failure with anemia or electrolyte disorders) and likely shared etiology (smoking with subsequent ischemic heart disease and lung disease). We similarly corroborated previously demonstrated clusters and high rates of comorbidity oriented around mental health in veterans^14,34–36^. We also identified unanticipated relationships, such as the presence of gout and skin ulcers resulting in a high relative risk for subsequent acute kidney failure. High dimensional analyses such as this one may generate hypotheses for future investigation of mechanistic explanations for the above unanticipated relationships.

The causal interpretations of this study are limited based on the data source, as EHR data are imperfect and certain diagnoses may be systematically misreported in routine clinical care. Additionally, the specific findings of our study are intended to provide knowledge within this fairly specific and homogeneous patient population and may not extrapolate to other populations. Moreover, veterans experience differing morbidities based on their service assignments which may impact the generalizability of findings even within the veteran population. These results are generated based on a single healthcare system; it is possible that veterans may have sought care outside the VA, despite its integrated nature. Computationally, certain decisions may impact the results. For instance, limiting the cohort to patients with 5-year EHR history to ensure a consistent level of follow-up for individual patients may have resulted in missed rapidly fatal events. Similarly, limiting diagnoses to those experienced by least 10% of patients may bias this analysis against rare diseases which may impact the network and its metrics. Larger longitudinal datasets will allow study of shorter follow-up times or rarer diagnoses. Despite these limitations, our study highlights fairly common and high impact diagnoses in an integrated practice setting.

This study focused on a highly selected cohort of overall healthy screening population patients. Future studies will focus on applying these algorithms to more heterogeneous and inclusive populations. Prospectively collected (though less granular) health status data are also available as a component of the study and we anticipate their use as a method for auditing the quality of EHR-based data.

## Methods

### Patient population and data sources

The CSP #380 cohort and the results of their baseline and surveillance colonoscopic exams have previously been described^25,37^. Briefly, 3,121 healthy veterans aged 50–75 underwent screening colonoscopy. Enrollment criteria included those with no lower gastrointestinal tract symptoms, prior history of colon disease, or a structural examination of the colon within 10 years. Exclusion criteria included medical conditions that would increase the risk of or preclude benefit of screening colonoscopy, including prosthetic heart valve, anticoagulant therapy, nonmedical social problems, need for special precautions (such as antibiotic prophylaxis), or being a woman of childbearing potential. In addition to specific data collected prospectively over the course of the 10-year study follow-up, all VA healthcare encounters from October 1999 to December 2015 were obtained from the VA Corporate Data Warehouse (CDW). The CDW includes EHR data such as inpatient and outpatient diagnoses (available as International Classification of Diseases, Ninth Edition [ICD-9] diagnosis codes), pharmacy data, and manually curated data for specific conditions. Of the 3,121 CSP #380 participants, we included only those with at least 5 years of follow-up in the VA CDW in this analysis.

All methods were carried out in accordance with relevant guidelines and regulations. The Durham Veterans Affairs (VA) Medical Center Institutional Review Board approved this secondary analysis under CSP #380 LA: Longitudinal Analysis of VA CSP #380 Screening Colonoscopy (MIRB # 1872). A waiver of informed consent has been granted by the Durham VA Medical Center Institutional Review Board for work performed under this protocol, including this secondary analysis.

### Data extraction and analysis

All VA inpatient and outpatient clinical encounters for CSP #380 participants were identified and extracted, including date of encounter and ICD-9 diagnosis codes. Analyses were performed in SAS version 9.4 (SAS Institute, Cary, NC) and R version 3.4.0 (R Foundation, Vienna, Austria). ICD-9 codes were collapsed into 3-digit codes and free text explanations were generated^38^. Three-digit codes were preferred over other alternatives due to their granularity (compared to major diagnostic categories) as well as their greater capture of comorbidities managed in the outpatient setting (compared to approaches such as diagnosis related groups). For the network analysis, diagnoses affecting fewer than 10% of patients were excluded, as were non-diagnostic V codes (Supplementary Classification of Factors Influencing Health Status and Contact with Health Services), complications of pregnancy, childbirth, and the puerperium (as women of childbearing potential were excluded from the study), symptoms, signs, ill-defined conditions (780.*-799.*), and unclassified complications of surgical and medical care (996.*-999.*). The first chronological instance of each diagnosis for each patient was identified.

The temporal relationship between each pair of three-digit ICD-9 diagnoses in a given patient was characterized. All pairs of diagnoses in the population were then aggregated in the form of an adjacency matrix of relative risks (RR). Each RR represents the ratio of the probability of developing a diagnosis *j* given a prior diagnosis *i* versus the probability of developing diagnosis *j* without a prior diagnosis *i* (Eq. 1). If diagnoses were coincident on the same day, these were excluded in the calculation (i.e. not in the numerator or denominator). The RR matrix was represented with each *i*th row and *j*th column. Thus, a RR > 1 represents a relationship between diagnoses where diagnosis *j* is more common in patients with a prior diagnosis *i* than in those without diagnosis *i*.

$$RR\left( {i,j} \right) = {\raise0.7ex\hbox{${\frac{{N\left( {i \to j} \right)}}{{N\left( {i \to j} \right) + N\left( {i \to \sim\,j} \right)}}}$} \!\mathord{\left/ {\vphantom {{\frac{{N\left( {i \to j} \right)}}{{N\left( {i \to j} \right) + N\left( {i \to \sim\,j} \right)}}} {\frac{{N\left( {\sim\,i \to j} \right) + N\left( {j \to i} \right)}}{{N\left( {\sim\,i \to j} \right) + N\left( {j \to i} \right) + N\left( {\sim\,i \& \sim\,j} \right)}}}}}\right.\kern-\nulldelimiterspace} \!\lower0.7ex\hbox{${\frac{{N\left( {\sim\,i \to j} \right) + N\left( {j \to i} \right)}}{{N\left( {\sim\,i \to j} \right) + N\left( {j \to i} \right) + N\left( {\sim\,i \& \sim\,j} \right)}}}$}}$$

### Equation 1. Relative risk calculation

#### Network features

iGraph was used to produce a directed graph of comorbidities with chronological RRs > 1^39^. The resulting directed graph was then analyzed for additional aggregate summary statistics. These included *edge density*, which is defined as the proportion of the number of directional relationships in the graph out of the total number of possible directional relationships (2^n^, where n represents the total number of diagnoses in the graph). *Reciprocity* was also assessed, representing the proportion of both diseases with RR > 1 out of all pairwise relationships. The *diameter* of the graph was also calculated using a breadth-first search like method, identifying the two diagnoses with the longest connection of diagnoses, demonstrating the longest path from a preceding to subsequent diagnosis^39^.

Key diagnoses were identified in the graph, including the diagnoses with the top scaled Kleinberg hub and authority centrality scores^40^. *Hub diagnoses* are preceding diagnoses with subsequent diagnoses of high “importance.” Conversely, *authority diagnoses* are subsequent diagnoses that are estimated to have high “importance” based on preceding diagnoses (Supplementary Figure S2). The *PageRank* of diagnoses within the network was also calculated^41^. The PageRank was the first algorithm used by Google and roughly estimates “importance” based on the number and importance of incoming links and is comparable to the authority centrality score.

The Infomap algorithm was used to identify communities of diagnoses in the network graph^42,43^. Communities represent groups of diseases that tend to be more densely connected with other diagnoses within the community compared to those outside the community. One hundred runs of the Infomap algorithm were used to partition the network^44^. Infomap utilizes the map equation, which is a flow-based and information-theoretic function. Minimizing the map equation across possible network partitions identifies regions in a network where a random walker tends to stay for a long time. While diagnoses within a single community may be connected with diagnoses in other communities, the identified communities overall have a greater flow together. For example, diagnoses outside a designated community may be connected to multiple diagnoses in the community but are excluded since they also have many connections to external diagnoses.

## Supplementary Information

Supplementary Information

## Data Availability

The aggregate data generated and/or analyzed during the current study are available from the corresponding author on reasonable request. Investigators (non-VA and VA) are invited to submit data and specimen requests for the Cooperative Studies Program #380 Cohort. The CSP 380 data dictionary is publicly available: https://www.research.va.gov/programs/csp/cspec/datadictionary_csp380.html.
