# Supplementary material for: Characterizing chronological accumulation of comorbidities in healthy veterans: a computational approach
Source: Sci Rep. 2021 Apr 14;11:8104. doi: 10.1038/s41598-021-85546-2 (PMC8046765; doi:10.1038/s41598-021-85546-2)
Supplement: Supplementary file 1 — Supplementary Information [file 41598_2021_85546_MOESM1_ESM.docx]

**Title:**

Characterizing chronological accumulation of comorbidities in healthy veterans: a computational approach

**Running title:**

Directed networks of comorbidities

**Authors:**

Julian C. Hong, MD, MS^1,2,3^

Elizabeth R. Hauser, PhD^1,4^

Thomas S. Redding, MS^1^

Kellie J. Sims, PhD^1^

Ziad F. Gellad, MD, MPH^1,5^

Meghan C. O’Leary, MA^1^

Terry Hyslop, PhD^1,4^

Ashton Madison, MPH^1^

Xuejun Qin, PhD^1,4^

David Weiss, PhD^6^

A. Jasmine Bullard, MHA^1^

Christina D. Williams, PhD, MPH^1,5^

Brian A. Sullivan, MD^1,5^

David Lieberman, MD^7,8^

Dawn Provenzale, MD, MS^1,5^

1. Cooperative Studies Program Epidemiology Center-Durham, Durham VA Health Care System, Durham, NC

2. Department of Radiation Oncology, University of California, San Francisco, San Francisco, CA

3. Bakar Computational Health Sciences Institute, University of California, San Francisco, San Francisco, CA

4. Department of Biostatistics and Bioinformatics, Duke University, Durham, NC

5. Department of Medicine, Duke University, Durham, NC

6. Perry Point VA Medical Center, Perry Point, MD

7. VA Portland Health Care System, Portland, OR

8. Oregon Health and Science University, Portland, OR

**Supplementary Table S1. Patient characteristics for those excluded with less than 5-year follow-up (n = 577)**

| **Variable** | **Number (%)/Median (IQR, range)** |
| --- | --- |
| Male | 564 (97.7%) |
|  |  |
| Age |  |
| At first diagnosis | 70.2 (65.1-74.7) |
| At last diagnosis | 72.8 (67.1-77.3) |
|  |  |
| Range between first and last diagnoses | 2.5 (1.0-3.8) |
|  |  |
| EHR ICD diagnoses per patient | 77 (27-166) |
| Number of distinct ICD diagnoses | 33 (14-56) |
| Number of distinct ICD three-digit diagnoses | 27 (12-43) |

*EHR, Electronic health record. ICD, International Classification of Diseases (ninth edition).*

**Supplementary Table S2. ICD 411.* (Other acute and subacute forms of ischemic heart disease) and highest relative risk subsequent diagnoses**

| ICD-9 | Diagnosis | Relative risk |
| --- | --- | --- |
| 410.* | Acute myocardial infarction | 2.50 |
| 425.* | Cardiomyopathy | 2.23 |
| 413.* | Angina pectoris | 2.13 |
| 584.* | Acute kidney failure | 1.87 |
| 403.* | Hypertensive chronic kidney disease | 1.86 |
| 426.* | Conduction disorders | 1.72 |
| 458.* | Hypotension | 1.70 |
| 412.* | Old myocardial infarction | 1.65 |
| 414.* | Other forms of chronic ischemic heart disease | 1.63 |
| 327.* | Organic sleep disorders | 1.57 |
| 280.* | Iron deficiency anemias | 1.57 |
| 511.* | Pleurisy | 1.55 |
| 428.* | Heart failure | 1.50 |
| 424.* | Other diseases of endocardium | 1.44 |
| 924.* | Contusion of lower limb and of other and unspecified sites | 1.45 |
| 585.* | Chronic kidney disease (CKD) | 1.41 |
| 433.* | Occlusion and stenosis of precerebral arteries | 1.40 |
| 287.* | Purpura and other hemorrhagic conditions | 1.40 |
| 436.* | Acute, but ill-defined cerebrovascular disease | 1.39 |
| 518.* | Other diseases of lung | 1.36 |

**Supplementary Table S3. Diagnoses with the highest relative risk of subsequent acute kidney failure (ICD 584.*)**

| ICD-9 | Diagnosis | Relative risk of acute kidney failure |
| --- | --- | --- |
| 401.* | Essential hypertension | 2.72 |
| 276.* | Disorders of fluid, electrolyte, and acid-base balance | 2.39 |
| 585.* | Chronic kidney disease (CKD) | 2.13 |
| 403.* | Hypertensive chronic kidney disease | 2.06 |
| 428.* | Heart failure | 2.02 |
| 411.* | Other acute and subacute forms of ischemic heart disease | 1.87 |
| 593.* | Other disorders of kidney and ureter | 1.83 |
| 274.* | Gout | 1.82 |
| 250.* | Diabetes mellitus | 1.79 |
| 425.* | Cardiomyopathy | 1.78 |
| 707.* | Chronic ulcer of skin | 1.72 |
| 285.* | Other and unspecified anemias | 1.72 |
| 458.* | Hypotension | 1.71 |
| 357.* | Inflammatory and toxic neuropathy | 1.65 |
| 440.* | Atherosclerosis | 1.61 |
| 429.* | Ill-defined descriptions and complications of heart disease | 1.60 |
| 427.* | Cardiac dysrhythmias | 1.56 |
| 424.* | Other diseases of endocardium | 1.52 |
| 682.* | Other cellulitis and abscess | 1.50 |
| 041.* | Bacterial infection in conditions classified elsewhere and of unspecified site | 1.49 |

**Supplementary Table S4. Diagnosis code communities**

| **ICD-9** | **Diagnosis** | **Cluster** |
| --- | --- | --- |
| 380.* | Disorders of the external ear | 1 |
| 294.* | Persistent mental disorders due to conditions classified elsewhere | 1 |
| 298.* | Other nonorganic psychoses | 1 |
| 331.* | Other cerebral degenerations | 1 |
| 436.* | Acute, but ill-defined, cerebrovascular disease | 2 |
| 438.* | Late effects of cerebrovascular disease | 2 |
| 435.* | Transient cerebral edema | 2 |
| 389.* | Hearing loss | 3 |
| 388.* | Other disorders of ear | 3 |

**Supplementary Figure S1: Directed network subgraphs based on ICD 411.* (Other acute and subacute forms of ischemic heart disease and acute kidney failure (ICD 584.*).** Subsequent and preceding paths based on highest relative risk (RR) for a major hub diagnosis, other acute and subacute forms of ischemic heart disease (left), and a major authority diagnosis, acute kidney failure (right).

### **Supplementary Figure S2: Hubs and authorities**
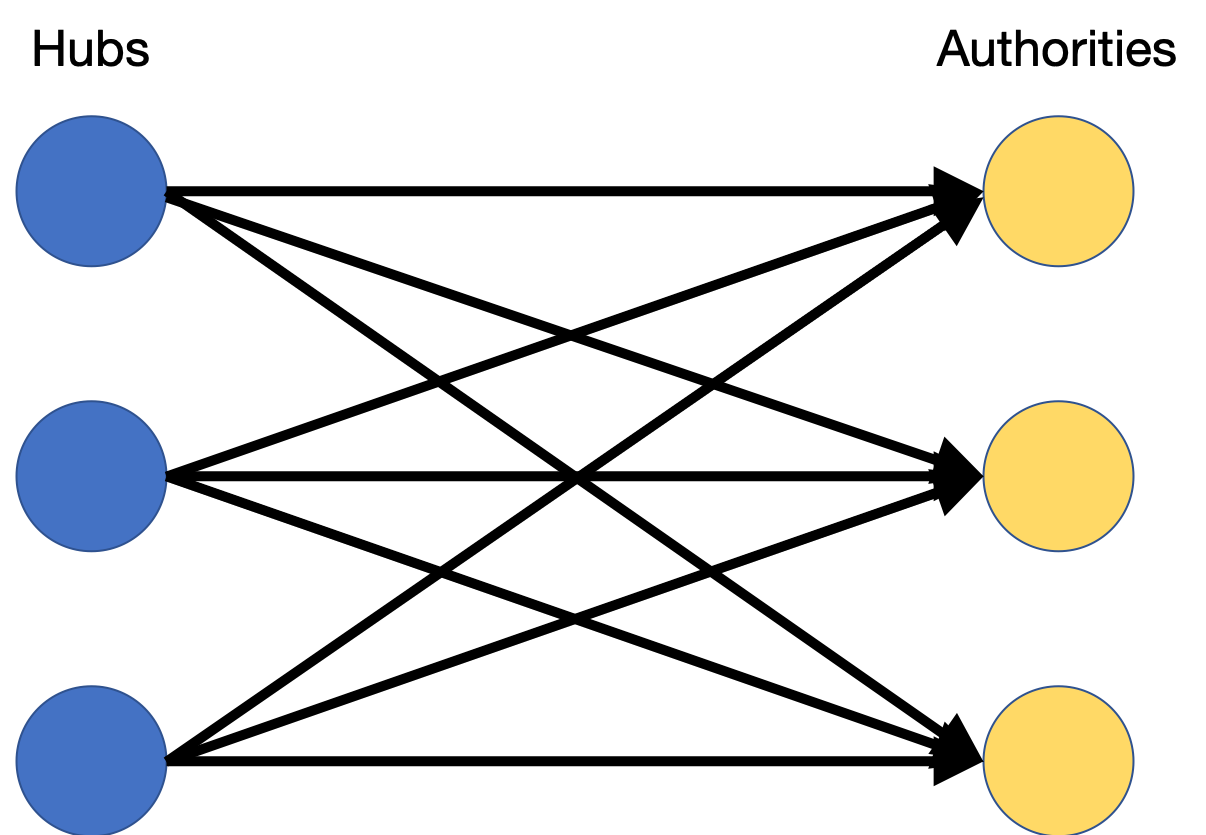


Hub diagnoses (blue) are preceding diagnoses with subsequent diagnoses of high “importance.” Conversely, authority diagnoses (yellow) are subsequent diagnoses that are estimated to have high “importance” based on preceding diagnoses.
